# Incidence rates and risk factor analyses for owner reported vomiting and diarrhoea in Labrador Retrievers – findings from the Dogslife Cohort

**DOI:** 10.1016/j.prevetmed.2017.02.014

**Published:** 2017-05-01

**Authors:** Carys A. Pugh, B. Mark de C. Bronsvoort, Ian G. Handel, Damon Querry, Erica Rose, Kim M. Summers, Dylan N. Clements

**Affiliations:** a Roslin Institute and Royal (Dick) School of Veterinary Studies, University of Edinburgh, Easter Bush, Midlothian, EH25 9RG, Scotland, United Kingdom; b Royal (Dick) School of Veterinary Studies and Roslin Institute, University of Edinburgh, Easter Bush, Midlothian, EH25 9RG, Scotland, United Kingdom

**Keywords:** LR, Labrador Retriever, Canine, Cohort, Vomiting, Diarrhoea, Epidemiology, Gastrointestinal

## Abstract

Dogslife collects data directly from owners of Labrador Retrievers across the UK including information regarding signs of illness irrespective of whether the signs precipitated a veterinary visit. In December 2015, the cohort comprised 6084 dogs aged up to six years and their owners had made 2687 and 2601 reports of diarrhoea and vomiting respectively. The co-occurrence of vomiting and diarrhoea with other signs was described and the frequencies and durations of the two signs were examined with reference to veterinary visitation. Age-specific illness rates were described and Cox Proportional Hazards models were used to estimate risk factors.

Just 37% of diarrhoea reports were associated with a veterinary visit and the proportion was even lower for vomiting at 28%; indicating that studies of veterinary practice data miss the majority of signs of gastrointestinal upset. In terms of frequency and duration, diarrhoea typically needed to last two days before the dog would be taken to the vet but if the dog vomited at least every six hours, the owner would be more likely to take the dog to the vet after one day.

The illness rates of both signs peaked when the dogs were aged between three and six months. There was also a seasonal pattern to the incidents with the lowest hazards for both in May. Diarrhoea incidents peaked in August-September each year but, while vomiting appeared to be higher in September, it peaked in February. Having another dog in the household was associated with a lower hazard for both vomiting and diarrhoea but having a cat was only associated with a reduced hazard of vomiting.

In addition to the distinct seasonal patterns of reporting, there were clear differences in the geographic risks for the two signs. The hazard of diarrhoea was positively associated with human population density within Great Britain (according to home post code) whereas no significant geographical association was found with vomiting.

This study is particularly relevant for dog owners because it highlights the wealth of gastrointestinal illnesses in dogs that are dealt with by owners but never seen by veterinarians. The risk factor analyses make use of owner-reported demographic information, highlighting the differences between vomiting and diarrhoea. The analyses give rise to the possibility that the presence of other pets in households may affect rates of illness and indicate new avenues for investigations of these distinct, and oft-suffered conditions.

## 1 Introduction

Traditionally, canine health data are collected at primary and secondary veterinary facilities. Case notes are routinely kept as part of providing care and it is advantageous to make use of them for epidemiological studies. In the UK, large-scale collection of veterinary records has been pioneered by the Small Animal Surveillance Network (SAVSNET) and VetCompass schemes. Both projects have the capacity to estimate the burden of specific conditions such as hyperadrenocorticism (O’Neill et al., 2016) or diarrhoea (Jones et al., 2014) and examine routinely collected data for potential risks. These data are generated by hundreds or thousands of veterinarians and inevitably lack consistency but both projects have attempted to standardise diagnostic criteria and are accumulating a vast wealth of data. However, risk factor analyses lack information regarding simple demographic factors such as household type or other pets in household and by definition, the data exclude signs of potential illness that do not precipitate veterinary visits. As such, they represent a sub-sample of signs seen by an owner.

Dogslife is a longitudinal prospective study of Labrador Retriever (LR) health in the UK (Clements et al., 2013, Pugh et al., 2015a, Pugh et al., 2015b). Contrary to most other studies, Dogslife gathers data via an online questionnaire directly from owners in a systematic and standardised way and this facilitates the collection of details of problems unreported elsewhere such as veterinary visits or insurance claims. The illness-related section of the Dogslife questionnaire (Appendix A: Illness questions) starts by asking the owner whether their dog has had any of a list of problems and, only if they answer ‘Yes’, does the questionnaire go on to ask whether they visited the vet. This distinctive approach offers a greater depth of health information that may be used to investigate disease aetiology. There is the potential that signs that do not precipitate veterinary visits may nevertheless be identified as risk factors for subsequent poor health. More broadly, by asking owners directly, analyses are particularly relevant to dog owners and their day-to-day experience of canine health which includes decisions about whether and when to take their dog to see a veterinarian. The study we report here takes owner reported data and uses it to describe the burden of vomiting and diarrhoea before undertaking risk factor analyses to try to understand what underlies the two conditions.

## 2 Methods

The study was approved by the Veterinary Ethical Review Committee of the University of Edinburgh.

Dogslife is an online longitudinal study of LR health in the UK. Recruitment of owners began in July 2010 and continues into 2017. Once registered, owners are prompted to repeatedly return to the Dogslife website to complete an online questionnaire about their dog’s lifestyle, morphology and health. Data were collected for this study via routine online reporting to the Dogslife project from July 2010 to December 2015. The dogs involved were all pedigree LRs registered with the UK Kennel Club (KC) and in December 2015, they had a maximum possible age of six years. Recruitment to the Dogslife project has previously been described by Clements et al. (2013). In brief, an automatic upload of all LRs newly registered with the UK KC was sent to Dogslife nightly. For owners who permitted their contact details to be shared with third parties such as Dogslife (56% by post and 49% by email), a postcard and/or email was sent asking them to register for the project at the Dogslife website using their dog’s date of birth and KC registration number. As such, all participants were registered LRs and came from a known sampling frame.

During registration, the owners were asked demographic questions including whether there were other pets in the household and their home postcode. Morphological, lifestyle and health information were then collected repeatedly via online questionnaire (the registration, owner demographics and dog purpose questions are available as Appendix B: Registration and demographics and the relevant sections of the illness part of the questionnaire are included as Appendix A: Illness questions). A system of automated emails and non-automated telephone reminders encouraged owners to report monthly when their dogs were under one year of age and quarterly thereafter. The questionnaire and data collected were validated via face-to-face visits and veterinary record assessment for a subset of the cohort (Pugh et al., 2015c). A description of the demographics, morphology, lifestyle and retention of the dogs up until three and a half years of age were reported by previously Pugh et al. (2015a). This 2015 study indicated that female owners were over-represented in the Dogslife cohort when compared to the owners of KC registered dogs in the UK but that the dogs were representative in terms of sex mix, coat colour and geographic distribution. The cohort was subject to disproportionate loss to follow up with multi-dog households more likely to remain in the study and family households more likely to drop out.

Each owner report of illness was reviewed by a veterinarian (DNC) and coded with presenting sign(s) and diagnoses using the VeNom coding system (VeNom Coding Group, 2016). A reported illness might have multiple presenting signs or diagnoses so signs that were reported to start within three days of each other were grouped as part of the same illness. For example, a dog that started vomiting on a Saturday evening and had diarrhoea on the Sunday would be considered to have one illness comprised of two presenting signs. All analyses here were based on the presenting signs rather than diagnoses because a presenting sign of diarrhoea is unambiguous but a diagnosis of giardiasis requires a veterinary visit and positive diagnostic test, and thus a higher diagnostic stringency. By using presenting signs, a greater number of incidents were available for analysis.

**Fig. 1:**
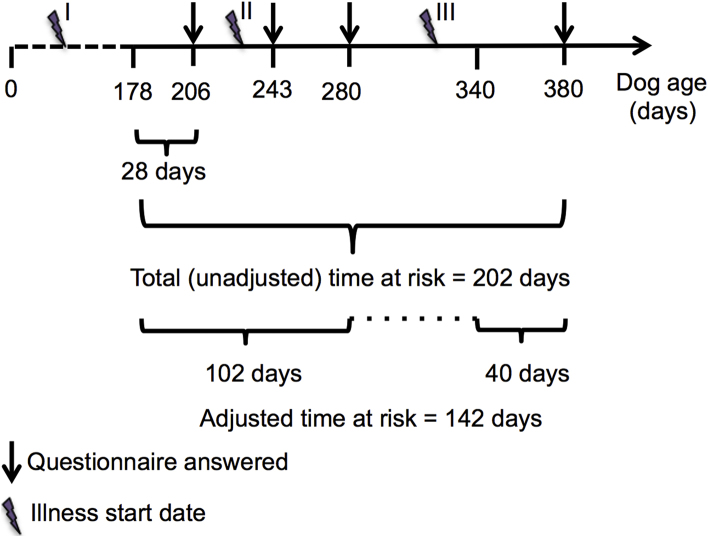
An example of the illness rate calculations for a single dog. The owner answered the questionnaire for the first time when the dog was 206 days old and most recently when the dog was 380 days old. The dog was ill three times and had an unadjusted rate = 2/202 and an adjusted rate 1/142 illnesses per dog day at risk. I: Illness included in count but excluded from all rate calculations II: Illness included in count and all rate calculations III: Illness included in count and unadjusted rate calculation only

All data tidying and analyses were undertaken using R (R Core Team, 2016). The reports of vomiting and diarrhoea were enumerated and described in the context of co-occurring signs. The rates of vomiting and diarrhoea in the cohort were calculated in two different ways. The first questionnaire for each dog asked about its health for the previous four weeks and subsequent iterations asked about the period since the owner last visited Dogslife. As such, the first total (unadjusted) rate was calculated using time at risk that began 28 days before the first questionnaire entry for each dog and ended with their most recent questionnaire answer. Illness dates were optional fields for the owners and suffered from missing data (typically for end dates). As such, in order to minimise the number of illnesses excluded due to lack of information, each illness event was considered to be instantaneous at the date of the first presenting sign for rate calculations. For the individual dog in Fig. 1 that suffered from three separate illnesses, only two would be included in the unadjusted rate calculation because the first one started more than 28 days before the owner answered the first questionnaire.

Validation work indicated a degree of recall decay in illness reporting for the cohort. When owners reported an illness that involved a veterinary visit to Dogslife, the median delay between visiting the vet and answering the Dogslife questionnaire was 16 days but this delay between illness and answering the questionnaire was 40 days when the owner did not report the illness to Dogslife (Pugh et al., 2015c). This was particularly relevant when considering risk in terms of season or month because if owners did not return to answer the questionnaire frequently, there was less confidence that the dog had truly been well for that period. Therefore, an adjusted time at risk was considered to start at 28 days before their first questionnaire answer but then only included periods of 40 days prior to each subsequent questionnaire answer. For the dog in Fig. 1, this would reduce the number of included illnesses to one and the time at risk would be adjusted to 142 days.

Illness rates were calculated separately for vomiting and diarrhoea and were also split according to age and month by dividing the number of events by the time at risk using the *survival* package in R (Therneau, 2014). Poisson confidence intervals (CIs) were calculated using the *poisson.test* command in the base *stats* package in R (R Core Team, 2016). The frequency and duration of reported signs were collated and compared with the proportion that precipitated a veterinary visit.

Modeling of time to all illness events included in the adjusted time at risk was approached by applying repeated events Cox Proportional Hazards models (Cox, 1972) using the *survival* package in R (Therneau, 2014). The models were checked by plotting time-varying estimates of the log of the hazard ratio for each parameter and the final model was selected on the basis of the robust score test for the whole model rather than at a 5% significance level for each variable considered. Dog identifiers were included as random variables but efforts to distinguish variance between owners and dogs by including owner as an additional random variable (relevant for owners with multiple dogs) indicated minimal difference so owner was dropped. The fixed variables considered were dog sex, dog purpose, coat colour, household type, whether there were cats or other dogs in the household, whether the owner smoked, the month each illness began, the age of the dog (as a binary variable using under one year and one year and above, and in age categories of zero to five years) and geographical variables discussed below. Interactions between age and month were considered.

Latitude and longitude were available for all dogs that had associated postcodes and associations between illnesses and continuous values of latitude and longitude were estimated. Human population density was available for postcode districts in Scotland, England and Wales but not Northern Ireland (NI) or many islands. Human population density was calculated as the number of people in the 2011 census (Office for Naitonal Statistics, [2011] for England and Wales and National Records of Scotland, [2011] for Scotland) divided by the area of the district in hectares and reported in 100 s of people per km^2^. Where population density, as a continuous variable, was significant in multivariable models, only Britain (GB, comprising England, Wales and Scotland) could be considered.

## 3 Results

On the 31st December 2015, the Dogslife cohort comprised 6084 dogs (3239 male, 2845 female), aged between 66 days and 2189 days (5 years, 11 months). At that time, the owners of 4728 dogs had completed at least one questionnaire (652 owners registered but did not start a questionnaire and 703 started but did not complete a questionnaire).

Retention for dogs aged over one, two, three, four and five years was 48.0%, 37.2%, 31.1%, 27.6% and 28.6% respectively, increasing to 62.1%, 48.9%, 40.%, 35.3% and 35.6% when owners that did not complete questionnaires were excluded (the apparent increase in retention for dogs over four and five years is due to changes in the denominator).

The owners of 4541 dogs gave valid UK postcodes and reported their smoking status and these dogs were used in later modelling. They comprised 2105 females and 2436 males and were black (2244), yellow (1225), chocolate (937) and fox red (135). In terms of purpose, the majority were pets (4100) but there were also working dogs (335), assistance dogs (43) and others (63). Their owners lived in England (3621), Scotland (664), Wales (181), NI (61) and Guernsey, Jersey and Isle of Man (14). The owners described their households as a family (1960), more than one adult (1953), retired (363), single adult (260) and not reported (5). Of these households, 1601 included at least one other dog, 987 included at least one cat and 741 included a smoker.

For the 4728 dogs with completed questionnaires, diarrhoea (VeNom code: Faecal appearance abnormal – diarrhoea) had been reported 2687 times. Of these reports, 37.4% (95% Confidence Interval (CI): 35.6–39.3%) involved a veterinary visit. In the majority of instances, diarrhoea was reported on its own but it was reported to co-occur with vomiting 606 times and with a number of other signs as shown in Table 1.

Vomiting (VeNom codes: Vomiting – other and Vomiting – haematemesis) was reported 2601 times but just 738 of those reports were associated with a veterinary visit (28.4%, 95% CI: 26.6–30.0%). Like diarrhoea, it was largely reported on its own but it was also reported to co-occur with other signs (Table 2, Table 3).

Both diarrhoea and vomiting were less likely to precipitate vet visits when occurring on their own (32.0% and 19.8% for diarrhoea and vomiting respectively) but when they were reported together, the dog was taken to the vet 49.5% (95% CI: 45.5–53.6%) of the time implying that owners were more concerned when the dog presented with more than one sign of illness.

The distribution of reports per dog is shown in Table 4. The majority of the dogs had just one report but there was one dog that was reported to vomit 14 times and another that was reported to have diarrhoea 12 times.

**Fig. 2:**
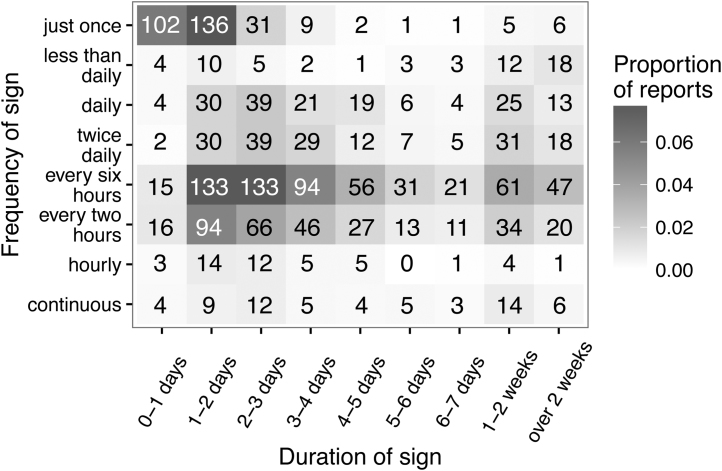
Duration and frequency of 1710 diarrhoea reports. The numbers of reports are given in each cell.

The questions regarding whether the dog had exhibited signs of illness were compulsory but subsequent questions regarding when the illness started and ended, whether the dog visited the vet and details of the frequency of the sign were all optional and therefore subject to some missing data. Of the 2016 reports of diarrhoea alone, 1710 had a start and an end date and a reported frequency. The frequency and duration of the diarrhoea is illustrated in Fig. 2 with a concentration of reports occurring either just once or happening every two to six hours and lasting one to three days. Interestingly, some owners who reported that their dog had diarrhoea just once simultaneously described the incident as lasting for more than one day (191 reports).

**Fig. 3:**
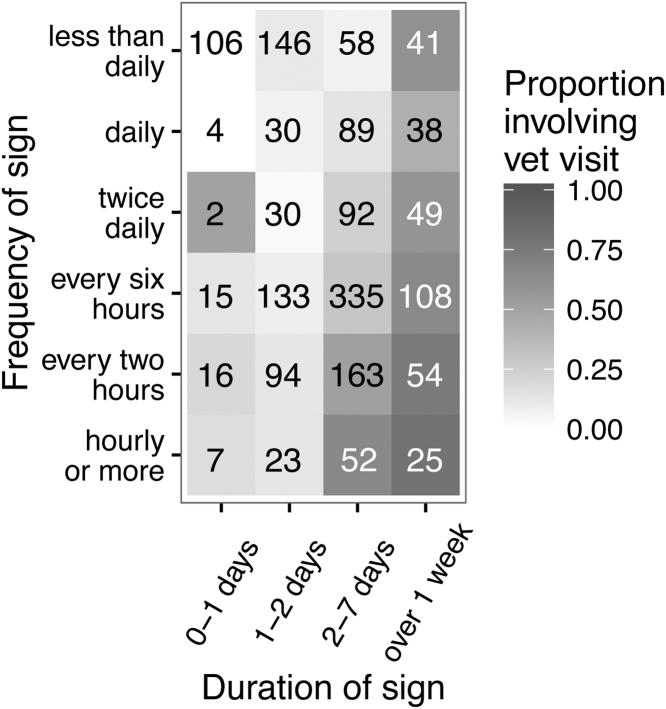
Proportion of 1710 diarrhoea reports that involve a veterinary visit according to frequency and duration of the sign. The catergory ‘just once’ has been included in the category ‘less than daily’. The denominators for the proportions are given in each cell.

The relationship between frequency, duration and proportion of reports that involved a veterinary visit is shown in Fig. 3. Broadly speaking, the more frequently the dog had diarrhoea or the longer the diarrhoea lasted, the higher proportion of the incidents involved a veterinary visit.

**Fig. 4:**
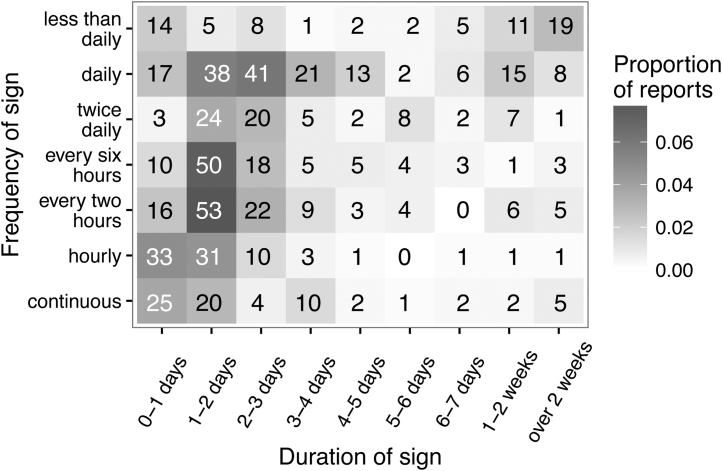
Duration and frequency of 669 vomiting reports (excluding the 1055 incidents that had a frequency of ‘once’). The exact numbers of reports are given in each cell.

**Fig. 5:**
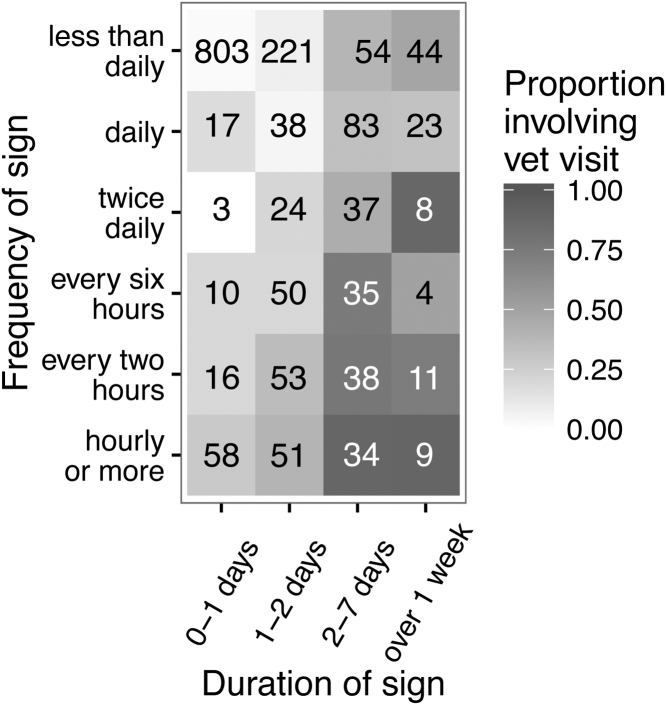
Proportion of 1724 vomiting reports that involve a veterinary visit according to frequency and duration of the sign (those with frequency ‘once’ are included in the ‘less than daily’ category). The denominator for each proportion is given in the relevant cell.

Considering just the 1901 reports of vomiting alone, 1724 had complete records for start and end dates and frequency of vomiting. The frequency and duration of these signs were dominated by reports where the dog vomited just once (45.8% of reports lasting less than one day, 12.5% of reports lasting 1–2 days). Fig. 4 therefore includes only the incidents where the dog was reported to vomit more than once (669 reports) and illustrates how they break down by duration and frequency. The relationship between frequency, duration and proportion of reports that involved a veterinary visit is shown in Fig. 5.

**Fig. 6:**
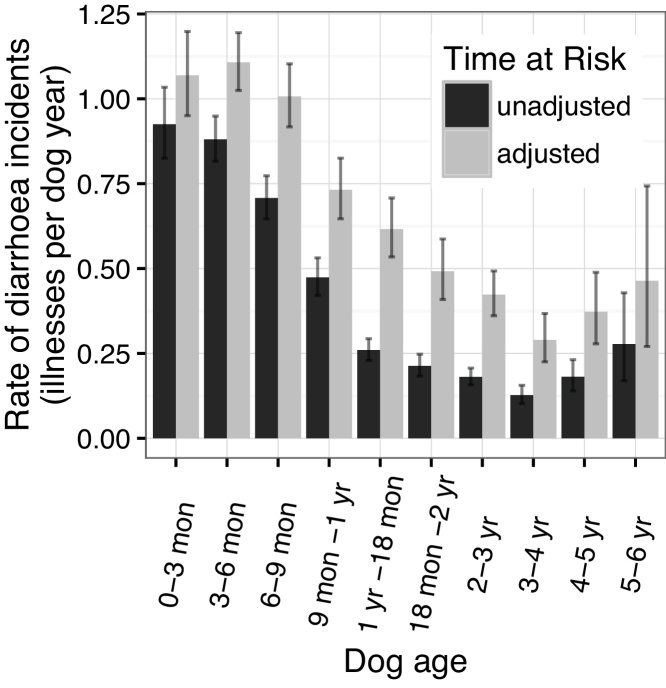
Rate of diarrhoea at different dog ages with 95% poisson confidence intervals.

**Fig. 7:**
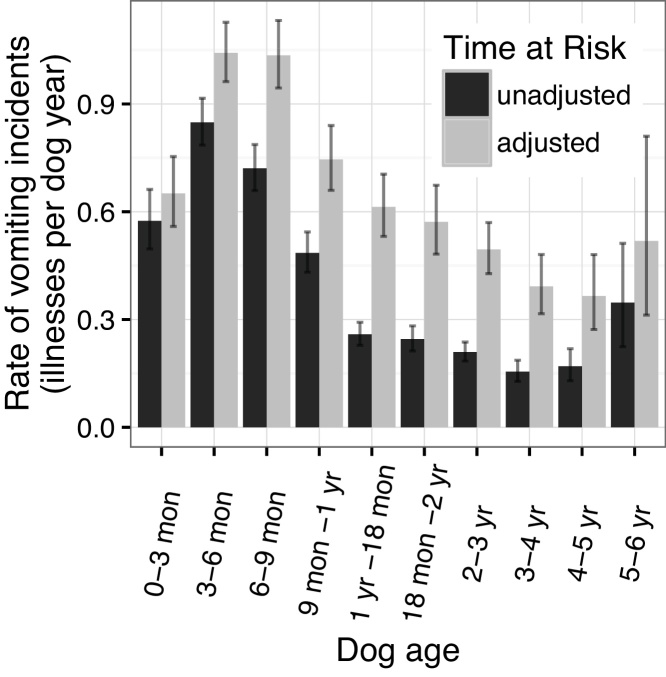
Rate of vomiting at different dog ages with 95% poisson confidence intervals.

The total time at risk was 6552.05 dog years and the adjusted time at risk comprised 3075.70 dog years. The rates of diarrhoea and vomiting were both strongly associated with the ages of the dogs (Fig. 6, Fig. 7). Both peaked when the dogs were between three and six months of age although the peak was more apparent for vomiting with a jump from 0.50 to 0.85 incidents per dog year (unadjusted) between the under three month and the three to six month age groups.

**Fig. 8:**
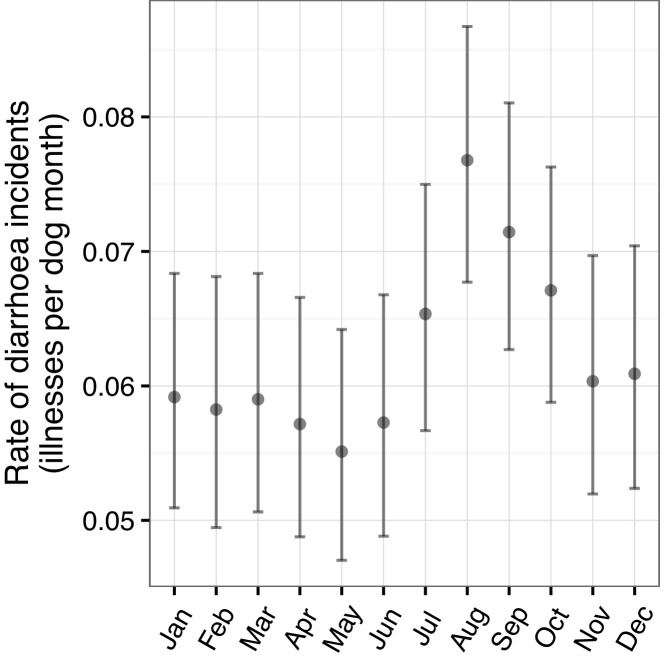
Monthly rates of diarrhoea with 95% poisson confidence intervals.

**Fig. 9:**
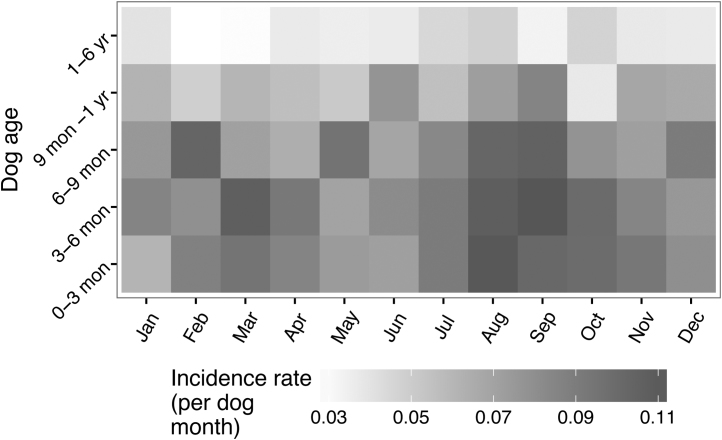
Monthly rates of diarrhoea split according to the ages of the dogs.

There were seasonal patterns of incidence for both diarrhoea and vomiting. Diarrhoea in particular appeared to peak in the summer months with the highest rate in August (Fig. 8) but this seemed to be particularly associated with the younger dogs (Fig. 9).

**Fig. 10:**
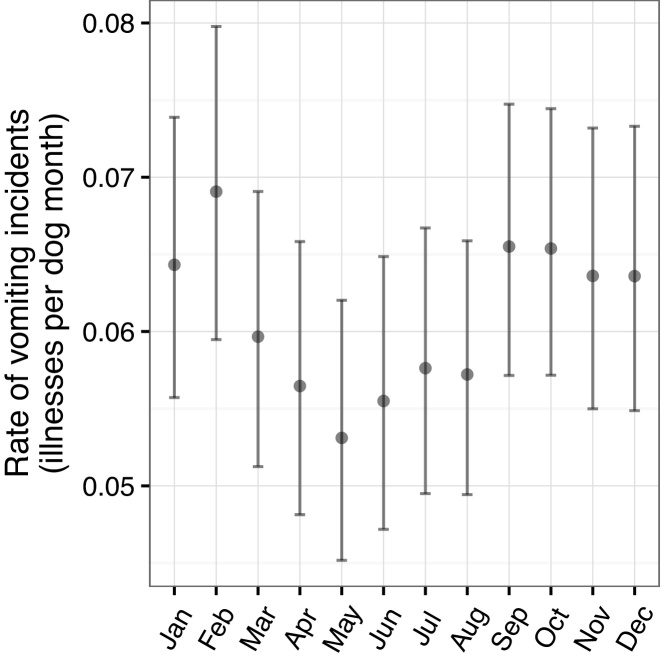
Monthly rates of vomiting with 95% poisson confidence intervals.

**Fig. 11:**
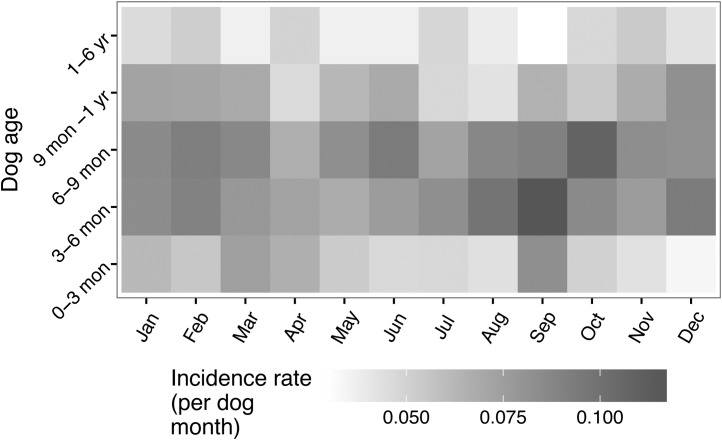
Monthly rates of vomiting split according to the ages of the dogs.

By contrast, Fig. 10 shows higher rates of vomiting toward the beginning of the year in addition to late summer with an overall lull in May. Again, the variation seems to be associated with the age of the dogs as can be seen in Fig. 11, with a peak in September for dogs under six months of age.

Table 5 includes the results of a Cox proportional hazards model of time to diarrhoea events in the cohort. Whether the dog was a pet, working or assistance dog was not found to be associated with time to diarrhoea incidents, nor was whether or not the owner also owned a cat. The sex of the dog was initially included in the model but it was also not associated with time to diarrhoea events.

By contrast, the factors associated with time to vomiting in the cohort did include owning a cat (Table 6) and working dogs had a hazard ratio of approximately half that of pets. The models are not directly comparable as the final vomiting model included all of the UK and the islands whereas human population density was a risk factor for diarrhoea so the model only includes the dogs in mainland GB. However the fact that population density was not a consistent risk in the two models is suggestive of different aetiologies for the two presenting signs. It was also interesting to note that the differences seen in the seasonal patterns in Fig. 8, Fig. 10 were maintained in these multivariable models. In both cases, the lowest hazards were seen in May but the higher hazards were in August for diarrhoea and February for vomiting.

## 4 Discussion

In the first five and a half years of data collection by Dogslife, both vomiting and diarrhoea were reported over 2600 times including 606 times together. Such large numbers of incidents allowed for in-depth analysis and comparison of the two signs. Only 37% of diarrhoea reports were associated with a veterinary visit but this was a third more than that of vomiting at just 28%. Both were considerably higher than the values from a study of 772 dogs in the UK in 2007 that found that 10% of dogs that had diarrhoea and 5% of dogs with vomiting were taken to the vet (Hubbard et al., 2007). The earlier study asked participants about the two weeks following receipt of the questionnaire so there is a possibility that different methodology might be affecting results. In particular, recall decay in Dogslife might differentially be affecting illness reports; incidents with perceived lower severity (such as those that do not precipitate a veterinary visit) have been found to be more likely to be forgotten compared to more serious illness events (US Department of Health Education and Welfare, 1961) so perhaps owners were forgetting to report the non-vet-visiting incidents of diarrhoea and vomiting to Dogslife.

There was presumably greater concern on behalf of owners when the two signs co-occurred because, when they were combined, owners took their dog to the vet in relation to 50% of events. Jones et al. (2014) studied events of diarrhoea that were presented to veterinary practices and they described a similar situation whereby dogs with uncomplicated diarrhoea (diarrhoea alone) had suffered the condition for longer before being presented to the vet than those dogs with multiple signs (complicated diarrhoea). This phenomenon has also been described in human health with a positive linear relationship between the concurrent number of possible signs and likelihood of visiting a GP (Elnegaard et al., 2015). In Dogslife, duration of sign rather than frequency appeared to be the primary motivation for veterinary visits when the dog had diarrhoea but there was more of an emphasis on frequency for vomiting. The Hubbard et al. (2007) study found that if a diarrhoea incident lasted for two of more days, 66% of reports involved a veterinary visit but they did not have any reports of vomiting that lasted more than two days. Interestingly, as reported to Dogslife, diarrhoea predominantly needed to last at least two days before the owner would take their dog to the vet but, for vomiting incidents that were happening at least every two hours, the owner would take their dog to the vet after just one day. Despite vomiting precipitating a lower proportion of veterinary visits than diarrhoea, if vomiting was frequent enough, owners would apparently react more quickly.

Both vomiting and diarrhoea had incidence rates that peaked between three and six months of age (adjusted rates 1.11 [95% CI: 1.02–1.19] and 1.04 [0.96–1.13] incidents per dog year for diarrhoea and vomiting respectively). Many factors would likely reduce the number of incidents in dog under three months of age. They initially consume a diet of the dam’s milk and would be partially protected by maternal immunity. Before completing early courses of vaccinations at approximately 12 weeks of age, owners are advised not to expose their puppy to other animals (Kennel Club, 2015) and, as such, the dogs would be exposed to fewer infectious agents. A study of shelter dogs in the US found that dogs under six months of age were disproportionately likely to suffer from diarrhoea when compared to dogs over six months of age (Tupler et al., 2012). Previous studies of multiple dog breeds in the UK considered dogs under one year as a single group so they could not identify this variability but they also found higher morbidities in dogs under one year when compared to older dogs (Batchelor et al., 2008, Jones et al., 2014).

Relative youth also appears to be associated with a seasonal pattern of reports, which may itself be related to a seasonal pattern of births (data not shown). For dogs under one year, the lowest incidence rates for both vomiting and diarrhoea occurred in May and there were peaks in late summer – August for diarrhoea and September for vomiting. However there were also distinct differences, including a high incidence rate for vomiting in February. It is possible that during the warmer, wetter months in the UK, the dogs are outside more and that infectious agents may better survive and thrive in the environment leading to a higher infectious risk, but this cannot explain the peak for vomiting in February. Batchelor et al. (2008) previously demonstrated a seasonal pattern of oocyst shedding in their study of diarrhoea in dogs in the UK so a seasonal pattern was not unexpected but the seasonal pattern of diarrhoea and vomiting incidence for Dogslife does not precisely accord with findings from a study of four large breeds in Norway (Sævik et al., 2012). The Norwegian study found peaks in Summer (June – August) for both signs at all ages and suggests that the peaks (with the exception of vomiting in February) may be driven by climactic factors, which would clearly differ between the two studies and countries.

Multiple studies have found higher rates of diarrhoea in males compared to female dogs (Hubbard et al., 2007, Sævik et al., 2012, Stavisky et al., 2011). It is interesting that despite having greater numbers of dogs than all of these studies (two of which were also based in the UK), there was no significant difference found between male and female Dogslife dogs. This analysis was based on 5426 dogs aged up to six years and male dogs appeared to succumb when younger than females but not significantly at the 5% level (results not shown). It should be noted that all of the other studies had a wider range of ages and breeds in their studied populations so it is possible that Dogslife LRs simply did not have a sex difference in terms of diarrhoea risk during the age range studied.

Direct comparison of the two multivariable models is hampered by the exclusion of dogs from Northern Ireland and the islands from the final model of diarrhoea meaning the models refer to slightly different populations. However they were built in the same way from the same initial population so the different findings emphasise the differences in the two presenting signs. In particular, there appears to be a different geographical component to the hazards. For example, human population density in GB was positively associated with diarrhoea but not with vomiting. Instead, the hazards from vomiting were associated with the countries within the UK where the dogs lived. Greater human population density would, on average, be associated with a greater number of potential infectious contacts. Diarrhoea has been associated with a number of infectious agents (Hackett and Lappin, 2003, Parsons et al., 2010) and one might hypothesise that more of the diarrhoea reports than the vomiting had an infectious aetiology.

The Norwegian study of four large breeds (Sævik et al., 2012) previously demonstrated that dogs in urban areas were at greater risk of developing diarrhoea than those in suburban or rural areas but they found no association for vomiting. Unfortunately Scotland uses a different method for describing urban/rural distinctions than NI and they are both different to the system used by England and Wales so assessing urban and rural risks was impractical with Dogslife data. Nevertheless, urban/rural classifications are typically based on human population density and the findings associating diarrhoea with higher levels of human population density seem to support the findings of the Norwegian study. The vomiting results were less clear-cut. Overall, the model fit was better when the different nations in the UK were included but the differences between the nations were not obvious. As such, it does not contradict the findings of the Norwegian study that also saw no effect.

In both models, the hazards for dogs from households of retired people were lower than those from the households comprising more than one adult. As with any analysis of owner-reported data, it is unclear whether this association was due to a true difference in risk between different household types or whether there were differences in reporting between household types. It is reassuring to note that family households had a reduced hazard of vomiting whereas that was not seen for diarrhoea indicating that, if differential reporting played a role, differences could still be seen been the two presenting signs.

The final differences between vomiting and diarrhoea were with regard to other pets in the household. Having a cat in the household was associated with a reduced hazard of vomiting reporting and having another dog was associated with a smaller hazard for vomiting than diarrhoea. One might imagine that having other pets would increase exposure to infectious disease so it is surprising that having another dog or a cat was associated with lower hazards. It is difficult to know whether these associations are genuine, perhaps relating to of more robust immune function, or whether the owners of these dogs are simply less likely to notice or report diarrhoea and vomiting.

Despite often being reported together, vomiting and diarrhoea were associated with different risk factors suggesting that the two signs have different aetiologies. On that basis, while there is clearly overlap between the conditions, future investigations of gastrointestinal disease should treat vomiting and diarrhoea as different processes or risk missing subtle sign-specific effects.

This study demonstrates the possibilities afforded by collection of owner-reported demographic, lifestyle and illness information. The findings extend analyses of gastrointestinal disease presented to veterinarians to include the majority of such illnesses, which are seen only by the owner. They give a unique picture of the burden of gastrointestinal disease experienced by young LRs in the UK and introduce the possibility that the purpose of the dog and the presence of other pets in households may affect rates of illness. As presenting signs, diarrhoea and vomiting can result from a wide range of causes and it appears that the risk factors associated with the conditions are equally complex.

## Figures and Tables

**Table 1:** Signs reported with diarrhoea and their reported frequency.

Other sign	Other sign	Reported frequency (percentage involving vet visit)
none	none	2016 (32.0)
Vomiting – other	none	606 (49.5)
Dietary indiscretion – other	none	25 (100)
Faecal appearance abnormal – haematochezia	none	10 (100)
Vomiting – other	Dietary indiscretion – other	7 (100)
Vomiting – other	Vomiting – haematemesis	6 (66.7)
Faecal appearance abnormal – haematochezia	Vomiting – other	5 (100)
Vomiting – haematemesis	none	4
Faecal appearance abnormal – haematochezia	Vomiting – haematemesis	4
Vomiting – other	Dietary indiscretion – other	2
Faecal appearance abnormal – other	none	2
Dietary indiscretion – foreign body ingestion	none	1
Dietary indiscretion – foreign body ingestion	Dietary indiscretion – other	1
Vomiting – haematemesis	Dietary indiscretion – other	1

**Table 2:** Signs reported with “Vomiting – other” and their reported frequency.

Other sign	Other sign	Reported frequency (percentage involving vet visit)
none	none	1902 (19.8)
Faecal appearance abnormal – diarrhoea	none	606 (49.5)
Dietary indiscretion – other	none	24 (91.7)
Vomiting – haematemesis	none	18 (11.1)
Dietary indiscretion – foreign body ingestion	none	8 (87.5)
Faecal appearance abnormal – diarrhoea	Dietary indiscretion – other	7 (100)
Faecal appearance abnormal – diarrhoea	Vomiting – haematemesis	6 (66.7)
Faecal appearance abnormal – diarrhoea	Faecal appearance abnormal – haematochezia	5
Faecal appearance abnormal – diarrhoea	Dietary indiscretion – foreign body ingestion	2
Faecal appearance abnormal – haematochezia	none	1
Vomiting – haematemesis	Dietary indiscretion – other	1

**Table 3:** Signs reported with “Vomiting – haematemesis” and their reported frequency.

Other sign	Other sign	Reported frequency (percentage involving vet visit)
none	none	14 (50.0)
Faecal appearance abnormal – diarrhoea	none	4
Faecal appearance abnormal – diarrhoea	Faecal appearance abnormal – haematochezia	2
Faecal appearance abnormal – diarrhoea	Dietary indiscretion – other	1

**Table 4:** Summary of the number of times each dog was reported to have diarrhoea or vomiting.

Number of incidents per dog	1	2	3	4	5	6	7	8	9	10	11	12	14
Diarrhoea (number of dogs)	1117	362	135	51	19	10	5	2	1	1	0	1	0
Vomiting (number of dogs)	943	291	149	57	32	12	12	4	2	1	1	0	1

**Table 5:** The results of a multivariable analysis of time to diarrhoea reports using a Cox proportional hazards model.

Variable	Hazard Ratio	95% Confidence Interval		Z score P value
		lower	upper	
**Other dog**				
No	–	–	–	–
Yes	0.78	0.71	0.87	**<0.001**
**Owner smoker**				
No	–	–	–	–
Yes	0.85	0.74	0.98	**0.023**
**Household type**				
More than one adult	–	–	–	–
Family	0.92	0.83	1.02	0.10
Retired	0.71	0.61	0.84	**<0.001**
Single adult	0.95	0.78	1.15	0.57
Not reported	0.57	0.20	1.60	0.28
**Longitude** (degrees)	1.02	0.99	1.06	0.13
**Latitude** (degrees)	0.97	0.94	1.00	0.09
**Density**^a^	1.01	1.00	1.01	**<0.001**
**Month**				
January	1.07	0.87	1.32	0.50
February	1.07	0.86	1.32	0.55
March	1.06	0.86	1.31	0.59
April	1.03	0.83	1.28	0.80
May	–			–
June	1.05	0.84	1.31	0.68
July	1.15	0.94	1.42	0.18
August	1.36	1.13	1.64	**0.001**
September	1.26	1.04	1.53	**0.02**
October	1.16	0.95	1.41	0.16
November	1.06	0.86	1.32	0.57
December	1.08	0.87	1.34	0.47

a Human population density (per 100 people per km^2^) according to postcode. Only available for mainland GB so model exclude dogs from Northern Ireland, Isle of Man, Jersey and Guernsey.

**Table 6:** The results of a multivariable analysis of time to vomiting reports using a Cox proportional hazards model.

Variable	Hazard Ratio	95% Confidence Interval		Z score P value
		lower	upper	
Other dog				
No	–	–	–	–
Yes	0.69	0.62	0.78	**<0.001**
**Cat**				
No	–	–	–	–
Yes	0.86	0.76	0.98	**0.02**
**Dog purpose**				
Pet	–	–	–	–
Assistance	0.89	0.51	1.54	0.67
Working	0.48	0.35	0.65	**<0.001**
Other	0.62	0.39	0.99	**0.04**
**Household type**				
More than one adult	–	–	–	–
Family	0.89	0.80	1.00	**0.05**
Retired	0.60	0.48	0.75	**<0.001**
Single adult	0.83	0.66	1.04	0.10
Not reported	0.81	0.36	1.85	0.62
**Location**				
England	–	–	–	–
Islands^a^	1.75	0.87	3.53	0.12
Northern Ireland	0.92	0.56	1.52	0.74
Scotland	0.88	0.75	1.04	0.13
Wales	0.91	0.70	1.19	0.49
**Month**				
January	1.17	0.95	1.43	0.13
February	1.27	1.04	1.55	**0.02**
March	1.09	0.89	1.34	0.42
April	1.04	0.84	1.29	0.70
May	–	–	–	–
June	1.05	0.84	1.30	0.67
July	1.09	0.88	1.34	0.43
August	1.08	0.88	1.31	0.47
September	1.20	0.99	1.46	**0.06**
October	1.19	0.97	1.44	0.09
November	1.17	0.95	1.43	0.14
December	1.14	0.93	1.40	0.20

a Jersey, Guernsey and Isle of Man.

## Appendix A Illness questions (© University of Edinburgh).

**Has Dog* had any of the following problems since you last visited the site?**

– Vomiting [Yes/No]
– Diarrhoea [Yes/No]
– Coughing [Yes/No]
– Scratching themselves [Yes/No]
– Licking or chewing themselves [Yes/No]
– Limping or lameness [Yes/No]

**Did Dog have any other illnesses or problems?** [Yes/No]

If ‘Yes’, **What was the problem?** (Optional question)

[Free text box]

All further questions are optional.

If ‘Yes’ to any, for each selection,

**Approximately when did the [insert illness name] start?**

[Pop-up calendar]

**Approximately when did the [insert illness name] get better?**

[Pop-up calendar]

**If your dog is not better yet, click the box below.**

**Approximately how often did the [insert illness name] happen?**

[Once/Continuous/Every hour/Every 2 h/Every 6 h/Every 12 h/Once a day/Once every 2 days/Once every 3 days/Once weekly/Once every two weeks/Once a month]

**Did you take Dog* to the vet for the [insert illness name]?**

[Yes/No]

If ‘No’ (no veterinary visit), **Do you know why Dog* developed this problem?** [Free text box]

If ‘Yes’ (veterinary visit), **Approximately when did you visit the vet?**

[Pop- up calendar]

*For Dog, each individual dog’s name is automatically inserted.

## Appendix B Relevant sections of Dogslife registration process and questionnaire (© University of Edinburgh).

1 Register your dog

*Required information

Your dog’s name *

(the name you call your dog rather than their registered Kennel Club name)

[free-text box]

What colour is your dog? *

[dropdown list of options: Black, Yellow, Chocolate, Fox Red, Other. If ‘Other’ is selected, a free-text box appears to state colour]

What sex is your dog? *

[select Male/Female]

What is your dog’s date of birth? *

[free-text box with calendar pop-up, and help button with image of Kennel Club certificate circling where to find it on the form]

Please confirm your dog’s date of birth? *

[free-text box with calendar pop-up]

Is your dog a Labrador Retriever? *

[select Yes/No]

What is your dog’s Kennel Club Registration Number (2 characters followed by eight digits) *

[free-text box, and help button with image of Kennel Club certificate circling where to find it on the form]

Confirm your dog’s Kennel Club Number *

[free-text box]

Users need to click Save & Continue to proceed to next page

2.1 Your Registration Details

*Required information

E-mail Address*

[free-text box]

Confirm e-mail Address*

[free-text box]

Please tick this box if you would prefer us **not** to contact you about your pet by e-mail:

[box to tick]

Please tick this box if you would prefer **not** to receive the Dogslife newsletter on a monthly basis:

[box to tick − if ticked next question disappears]

Please indicate which format you would like to receive the newsletter in:

[select Plain text/HTML]

Password (between 6 and 11 characters)

[free-text box]

Confirm Password

[free-text box]

Users need to click Save & Continue to proceed to next page

2.2 Your Profile

*Required information

Title*

[dropdown list: Mr, Mrs, Miss, Ms, Dr, Professor, Other. If ‘Other’ is selected, a free-text box appears to state title]

First Name*

[free-text box]

Surname*

[free-text box]

Daytime Contact Telephone Number (including STD code)*

[free-text box]

Please tick this box if you do **not** want us to contact you about your pet by telephone:

[box to tick]

Add second contact email? (optional)

Second e-mail

[free-text box]

Confirm second e-mail

[free-text box]

Users need to click Save & Continue to proceed to next page

2.3 Your Household

*Required information

Post Code (must be valid UK Post Code)*

[free-text box]

How would you describe your household? *

[dropdown list: Single Adult, More than one Adult, Family (one or more adult and one or more children), Retired (Single or Couple), Other. If ‘Other’ is selected, a free-text box appears to enter details]

Does anybody in the household smoke?*

[select Yes/No]

Are there other pets in the household?*

[select Yes/No. If ‘Yes’ selected question below appears]

What species and how many?

[dropdown list: Dogs, Cats, Other, and dropdown list of number. If ‘Other’ is selected, a free-text box appears. Option to add more so boxes appear again]

Users need to click Save & Continue to proceed to next page which reads:

**Thank you!**

Thanks for registering [dog name] with Dogslife.

Questionnaire

1.1 Pet Profile

*Required information

Do you own [dog name] primarily as a:*

[dropdown list: Household pet, Working dog (e.g. Gundog), Assistance Dog (e.g. Guide Dog), Other. If ‘Other’ selected, free-text box appears to enter details. This question should only be asked the first time the user completes the questionnaire, so should not appear the next times they complete the questionnaire]

Users need to click Save & Continue to proceed to next page
